# Dual-light emitting 3D encryption with printable fluorescent-phosphorescent metal-organic frameworks

**DOI:** 10.1038/s41377-023-01274-4

**Published:** 2023-09-12

**Authors:** Jin Woo Oh, Seokyeong Lee, Hyowon Han, Omar Allam, Ji Il Choi, Hyeokjung Lee, Wei Jiang, Jihye Jang, Gwanho Kim, Seungsoo Mun, Kyuho Lee, Yeonji Kim, Jong Woong Park, Seonju Lee, Seung Soon Jang, Cheolmin Park

**Affiliations:** 1 Department of Materials Science and Engineering, Yonsei University, Seoul, 03722 Republic of Korea; 2 The George W. Woodruff School of Mechanical Engineering, Georgia Institute of Technology, 801 Ferst Drive, Atlanta, GA 30332-0405 USA; 3 School of Materials Science and Engineering, Georgia Institute of Technology, 771 Ferst Drive, Atlanta, GA 30332-0245 USA; 4 Spin Convergence Research Center, Korea Institute of Science and Technology (KIST), Seoul, 02792 Republic of Korea

**Keywords:** Optical materials and structures, Imaging and sensing

## Abstract

Optical encryption technologies based on room-temperature light-emitting materials are of considerable interest. Herein, we present three-dimensional (3D) printable dual-light-emitting materials for high-performance optical pattern encryption. These are based on fluorescent perovskite nanocrystals (NCs) embedded in metal-organic frameworks (MOFs) designed for phosphorescent host-guest interactions. Notably, perovskite-containing MOFs emit a highly efficient blue phosphorescence, and perovskite NCs embedded in the MOFs emit characteristic green or red fluorescence under ultraviolet (UV) irradiation. Such dual-light-emitting MOFs with independent fluorescence and phosphorescence emissions are employed in pochoir pattern encryption, wherein actual information with transient phosphorescence is efficiently concealed behind fake information with fluorescence under UV exposure. Moreover, a 3D cubic skeleton is developed with the dual-light-emitting MOF powder dispersed in 3D-printable polymer filaments for 3D dual-pattern encryption. This article outlines a universal principle for developing MOF-based room-temperature multi-light-emitting materials and a strategy for multidimensional information encryption with enhanced capacity and security.

## Abstract (web summary)

High security solid-state optical encryption is developed, based on fluorescent and phosphorescent dual-light-emitting MOFs in combination with 2D- and 3D-printing technologies.

## Graphical abstract

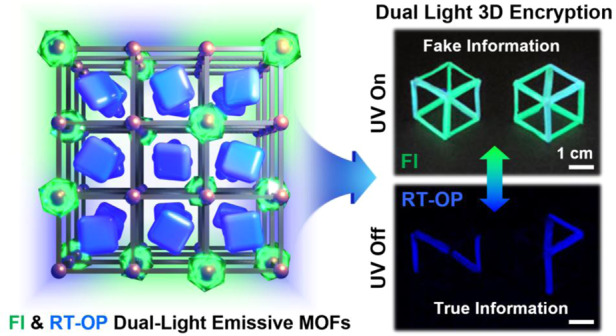

## Introduction

Over the past decade, cutting-edge technologies have witnessed rapid progress, completely transforming the manner in which people connect and communicate with one another, thereby realizing a “hyper-connected society”^1,2^. However, this connected society has caused gradual degradation in information security, including privacy, confidentiality, and secrecy, which is often cherished by individuals. Therefore, ensuring confidential data security based on an encryption system is of utmost importance^2,3^. Accordingly, information encryption technologies based on electrical or optical encryption have been widely developed^4^. Among these optical encryption technologies, including those based on chromism^5^, structural color^6^, and metasurface holography^7^, the technologies based on photoluminescence (PL) are of particular interest owing to their self-emitting capabilities, high brightness, and efficiencies, in addition to low probabilities of being hacked by digital computing systems^8^.

Although PL encryption technologies based on organic fluorescent materials are prevalent, room-temperature organic phosphorescence (RT-OP), which originates from the radiative transition of excitons from the triplet excited state to the ground state, has also garnered significant attention from numerous fields, such as display, bioimaging, document security, encryption, and anti-counterfeiting^4,9,10^. To achieve efficient and stable RT-OP, massive dissipation of the excitons via non-radiative processes which hampers efficient energy transfer should be minimized^4,9,11–13^. One of the most representative synthetic strategies for achieving stable RT-OP involves physically rigidifying a pair of host-guest molecules to ensure their close proximity for a stable energy transfer process^11,14^. Notably, various host-guest pairs designed via co-crystallization^15^, H-aggregation^16^, and supramolecular self-assembly^17^ have been successfully fixed in polymer matrices^10^, small molecular organic matrices^12,18^, metal-organic frameworks (MOFs)^19,20^, and inorganic matrices^21^. In some cases, additional rigidifying agents are required to minimize molecular vibrations and, thus, ensure efficient energy transfers^10,21,22^. However, the use of additives such as water^12,23,24^, metal ions^25^, and organic solvents^26^ to rigidify host–guest pairs often limits the solid-state applications of RT-OP materials.

Optical encryption based on the dual light emission of PL and RT-OP would allow unprecedented high-security level information protection especially when the emission of PL and RT-OP are independently controlled^27^. Notably, most dual PL and RT-OP emissive materials have been developed for efficient color mixing in display since RT-OP results from the singlet-to-triplet energy transfer of the excitons activated for PL^28,29^. In other words, the materials emitted both PL and RT-OP at the same time. Due to the longer lifetime of RT-OP than PL, the RT-OP is only visible when the incident exciting light source is turned off. Only a few works demonstrate the independent light emission of PL and RT-OP for high-security encryption^27^. We envisioned that MOFs classified as porous crystalline materials constructed using metal ions or metal clusters coordinated with organic ligands^30–33^ would be useful for realizing independent PL and RT-OP emission. Accordingly, when guest molecules for RT-OP were securely fixed within each periodic cavity of an MOF, the host-guest pairs were rigidified with fixed molecular separation, owing to the crystalline structure of the MOFs, resulting in stable RT-OP even without additives^14,34^. Furthermore, a rigidified host-guest MOF with Pb metal ions periodically coordinated in the MOF could be utilized as a template for synthesizing fluorescent Pb-based perovskite nanocrystals (NCs)^35^. This synthesis process leads to the creation of a novel dual-light-emitting MOF that demonstrates independent fluorescence and phosphorescence, making it suitable for information encryption with considerable capacity and security.

**Fig. 1:**
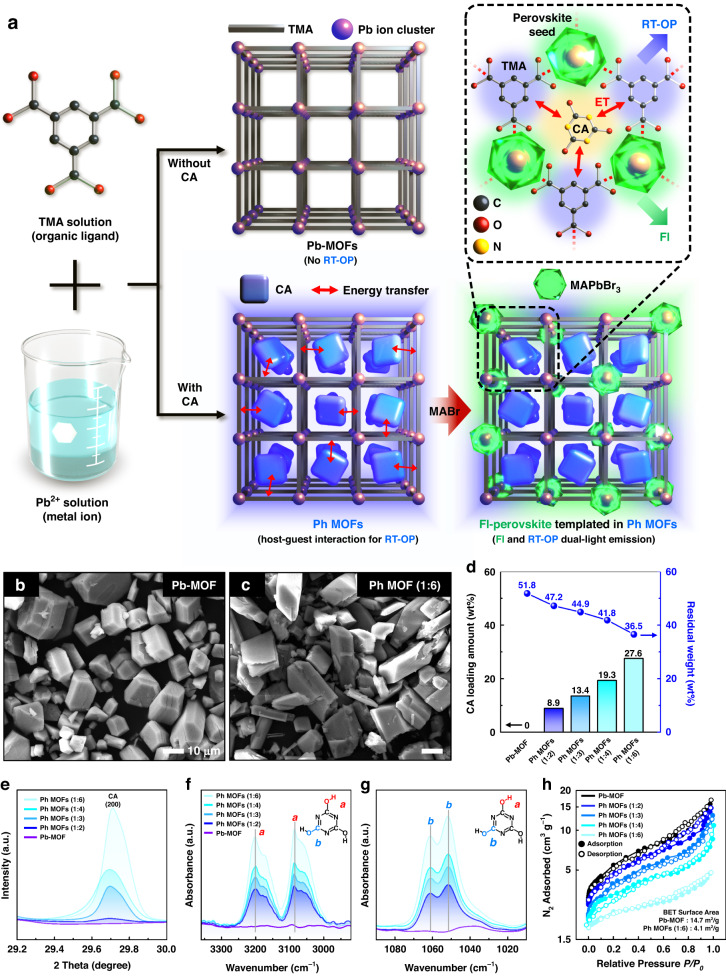
Synthesis and characterization of the MOF particles. **a** Schematic of the synthesis process for the Pb-MOF, Ph MOF, and Fl–Ph MOF samples. SEM images of the **b** Pb-MOF and **c** Ph MOF (1:6). **d** Actual CA loading (in percentage) of the Ph MOFs based on thermogravimetric analysis. **e** Normalized XRD peaks of the (200) plane of CA for the Pb-MOF and Ph MOFs with different CA loadings. **f** O–H bond and **g** C–O bond stretching vibrational peaks obtained from the FTIR spectra of CA, the Pb-MOF, and the Ph MOFs with different CA loadings. **h** N_2_ adsorption/desorption isotherms of the Pb-MOF and the Ph MOFs with different CA loadings at 77 K

With this background, herein, we present three-dimensional (3D) encryption with dual-light-emitting moldable and printable fluorescent–phosphorescent MOFs (Fl–Ph MOFs). Our Fl–Ph MOFs were based on Pb-containing MOFs with trimesic acid (TMA) organic ligands. The inclusion of cyanuric acid (CA) molecules as guests in the periodic cavities of the Pb-containing MOFs resulted in the efficient rigidification of both TMA and CA. This rigidification achieved a suitable distance for dexter energy transfer under ambient conditions, ultimately leading to stable deep-blue RT-OP. When a perovskite precursor, methylammonium bromide (MABr), was mixed with the Pb-containing MOF complexed with CA, fluorescent MAPbBr_3_ NCs exhibiting characteristic green emission under ultraviolet irradiation were successfully synthesized in the MOF, resulting in dual-light-emitting Fl–Ph MOFs, as illustrated in Fig. 1a. The powders of our Fl–Ph MOFs could be conveniently screen printed based on an art technique called “pochoir” for optical encryption. Typically, in an encrypted pattern, the actual information written with transient phosphorescence is hidden behind the fake information with green fluorescence under UV exposure. When the UV source is turned off, the fake information in green rapidly disappears, making the real information with long-lasting blue phosphorescence visible. Furthermore, a 3D structured cube-type skeleton was developed with the Fl–Ph MOFs dispersed in a moldable polymer matrix. Next, viewing-angle-dependent real and fake information in the Fl–Ph MOF-based cubes was successfully encrypted and deciphered using a smartphone software, facilitating the development of multiple high-security information encoding systems.

## Results

### Synthesis and characterization of the Ph MOFs

Notably, MOFs are crystalline materials composed of metal ions and organic ligands, and these materials have attracted considerable interest owing to their excellent thermal and chemical stability and capability of encapsulating a wide variety of guest species, including gases, nanoparticles, enzymes, and organic molecules, in their cavities^30,33,34^. Typically, the individual pores in MOFs can serve as a rigid matrix, and emitter molecules can be trapped in the solid pore cages for stabilization. In this study, we employed a Pb-based MOF (Pb-MOF) comprising coordinated Pb^2+^ metal centers bridged via TMA organic linkers. The Pb^2+^ centers in the Pb-MOF were used as the Pb ion sources for synthesizing fluorescent perovskite NCs. In addition, by introducing CA molecules into the cavities of the Pb-MOF, RT-OP resulting from dexter energy transfer between the rigidified CA and TMA in the framework was realized, as illustrated in Fig. 1a. Here, CA was added during the Pb-MOF synthesis to deposit CA molecules in the pores of the Pb-MOF. Note that the MOFs have the ability to fixate CA molecules with a constant molecular distance from the TMA molecules. This arrangement enables dexter energy transfer between the two species, namely CA (donor) and TMA (acceptor), leading to room temperature operation (RT-OP) without the need for additional rigidification agents like water^12^. Moreover, perovskite NCs were subsequently synthesized by reacting the CA-containing Pb-MOF with the MAPbBr_3_ precursor (that is, MABr in methanol). The resulting products, that is, CA-containing Pb-MOFs with MAPbBr_3_ NCs, emitted characteristic fluorescence and phosphorescence, attributed to the perovskite NC and host-guest energy transfer, respectively, as schematically presented in Fig. 1a. We hereafter denote the phosphorescent CA-containing Pb-MOFs as Ph MOFs.

Before the synthesis of MAPbBr_3_ NCs in Ph MOFs, we first investigated the properties of the CA-containing Pb-MOFs (Ph MOFs) with different CA loadings. For this, four types of Ph MOFs with initial CA loadings of 200, 300, 400, and 600% with respect to the TMA content (denoted as Ph MOF (1:2), (1:3), (1:4), and (1:6), respectively) were synthesized via the conventional solvothermal reaction of TMA, CA, and Pb ions in water at 63 °C for 1 h. The white solid product was washed three times with deionized (DI) water^35^. The white precipitates obtained after centrifugation were dried in a vacuum oven at 70 °C. The detailed synthetic procedures and precursor compositions of the CA-containing Pb-MOFs are presented in the Experimental Section. The scanning electron microscopy (SEM) images in Fig. 1b, c reveal that all the synthesized Ph MOFs with different CA contents consisted of triclinic crystals with an average longitudinal dimension of approximately 20 μm. The results indicate that the incorporation of CA in the Pb-MOFs barely altered the morphologies of the final materials compared with that of the pristine Pb-MOF (Fig. S1).

The amount of CA loaded into the Pb-MOF was estimated via thermogravimetric analysis under air (Fig. S2). The amount of CA loading increased with an increase in the CA concentration used in the synthesis process (Fig. 1d and Table. S1). The normalized residual weights of the samples decreased with increasing CA content owing to the increase in the organic moieties of TMA and CA in a given sample with a constant initial weight, as depicted in Fig. 1d. Based on the results obtained, the weight percentages of CA in the Ph MOF (1:2), (1:3), (1:4), and (1:6) samples were determined to be approximately 8.9, 13.4, 19.3, and 27.6 wt%, respectively (Fig. 1d). The powder X-ray diffraction (XRD) analysis of the Ph MOF series revealed that the characteristic diffraction peak at (220), arising from the triclinic Ph MOF crystals, intensified as the CA content in the Pb-MOF increased (Figs. 1e, S3). In the Fourier-transform infrared spectroscopy (FTIR) spectra of the Ph MOFs, the strong characteristic peaks of CA at 3087 and 1052 cm^−1^, corresponding to O–H and C–O, respectively, increased in intensity with increasing CA content, indicating that the CA content in the Pb-MOF could be systematically controlled (Figs. 1f, S4). Furthermore, N_2_ adsorption/desorption isotherms of the Pb-MOF and the Ph MOF samples with different CA loadings are illustrated in Fig. 1h; the surface area of the pristine Pb-MOF (approximately 14.7 m^2^ g^−1^) decreased following the incorporation of CA. The surface areas were approximately 13.8, 11.0, 9.4, and 4.1 m^2^ g^−1^ for the Pb-MOF (1:2), (1:3), (1:4), and (1:6) samples, respectively. These results clearly indicate that the cavities in the pristine Pb-MOFs were efficiently occupied by the CA molecules^32,34^.

### Photophysical properties of the Ph MOFs

**Fig. 2:**
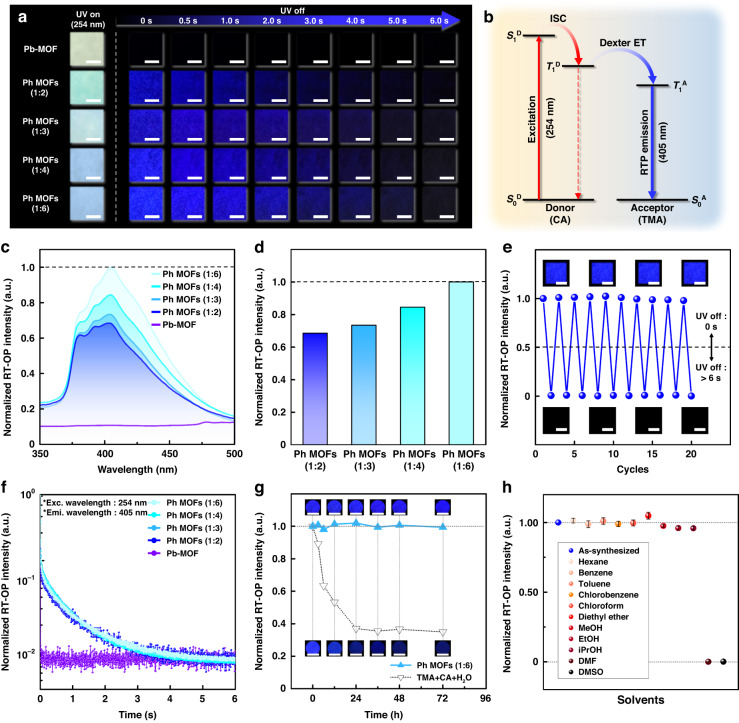
Photophysical properties of Ph MOFs at room temperature. **a** Photographs of the Pb-MOF and Ph MOFs with different CA loadings under UV light irradiation (254 nm) and after different intervals after turning off the UV lamp (scale bars: 1 cm). **b** Energy-transfer mechanism for the deep-blue (405 nm) phosphorescence of the Ph MOF at room temperature. **c** Steady-state RT-OP spectra and **d** normalized intensities of the 405 nm emission for the Pb-MOF and the Ph MOFs with different CA loadings. **e** RT-OP on/off switching cycle test with the Ph MOF (1:6) sample (scale bars: 5 mm). **f** Time-resolved phosphorescence decay curves of the Pb-MOF and the Ph MOFs with different CA loadings. **g** Phosphorescence intensities of the Ph MOF (1:6) and TMA/CA/H_2_O mixture exposed to an atmosphere with an RH of 10% over time (scale bars: 1 mm). **h** Stability of the phosphorescence intensity of the Ph MOF (1:6) sample in various solvents

The moderate deep-blue RT-OP emission of each Ph MOF powder sample could be clearly observed for over 3–4 s after the UV (wavelength: 254 nm) lamp was turned off, whereas the bare Pb-MOF presented no afterglow (Fig. 2a). The series of photographs for the four Ph MOF samples with different CA contents indicates that the intensity of the RT-OP emission increases with the CA content in the MOF, possibly owing to increased dexter energy transfer from CA to TMA^12^, as schematically illustrated in Fig. 2b. The PL spectra of the Ph MOF series in Fig. 2c reveal that the RT-OP intensity at the wavelength of 405 nm increased with the CA content, consistent with the visual observation. These results indicate that energy transfer between the CA and TMA species occurred successfully, indicating a sufficiently short distance between the CA and TMA units in the molecular framework, without additional rigidifying agents^12^. The normalized RT-OP intensities of the samples displayed in Fig. 2d reveal that Ph MOF (1:6) had the highest RT-OP intensity. Moreover, the absolute phosphorescence quantum yield of Ph MOF (1:6) is approximately 49.4% in air at room temperature. A further increase in the CA content in the Ph MOF, however, resulted in the precipitation of the excess CA due to the limited solubility of CA. No improvement of its phosphorescent properties was observed (Fig. S5). Remarkably, the phosphorescence of Ph MOF (1:6) was stable and reliable. The degradation in the RT-OP of the Ph MOF (1:6) sample was barely observable after more than 20 UV on/off cycles (Fig. 2e). The characteristic phosphorescence decay behaviors of the four Ph MOF samples (Fig. 2f) revealed that the average lifetimes (*τ*_ave_) of the Ph MOF powders with different CA contents were similar at approximately 0.52 s^12^.

The RT-OP stability of Ph MOF (1:6) was examined under continuous UV exposure, and the corresponding results are presented in Fig. 2g. In previous studies, the phosphorescence resulting from the dexter energy transfer between CA and TMA was triggered via water-driven hydrogen-bonding between the hydroxyl groups of CA and carboxyl groups of TMA, and the phosphorescence degraded with water evaporation^12,22^. To exclude the possible contribution of water in Ph MOF (1:6), the stability of its RT-OP was examined under a low relative humidity (RH) of approximately 10%. For comparison, a mixture of TMA and CA with H_2_O was prepared. The Ph MOF (1:6) sample maintained its initial RT-OP intensity over 3 days upon exposure to an atmosphere with 10% RH, whereas the RT-OP intensity of the TMA/CA/H_2_O mixture declined rapidly as H_2_O evaporated over time (Fig. 2g). These results corroborate our speculation that the efficient RT-OP emission of the Ph MOF is attributable to its uniform and rigid framework capable of holding CA close to TMA without water.

The solvent stability of our Ph MOF (1:6) sample was examined by observing its RT-OP when immersed in a variety of solvents (Fig. 2h and Supplementary Video 1). The Ph MOF (1:6) sample remained intact, and its initial RT-OP intensity was maintained over time when immersed in hexane, benzene, toluene, chlorobenzene, chloroform, diethyl ether, methanol, ethanol, and isopropanol, which are poor solvents for CA. However, the RT-OP of the Ph MOF (1:6) sample was lost after immersion in good solvents for CA, such as dimethylformamide (DMF) and dimethyl sulfoxide (DMSO). This loss indicates that CA became physically separated from TMA and was released from the Pb-MOF, ultimately resulting in the loss of the RT-OP emission of the sample^12^. The effect of the solvent was also confirmed based on the change in the XRD pattern (Fig. S6).

### Fl–Ph MOFs with perovskite NCs

**Fig. 3:**
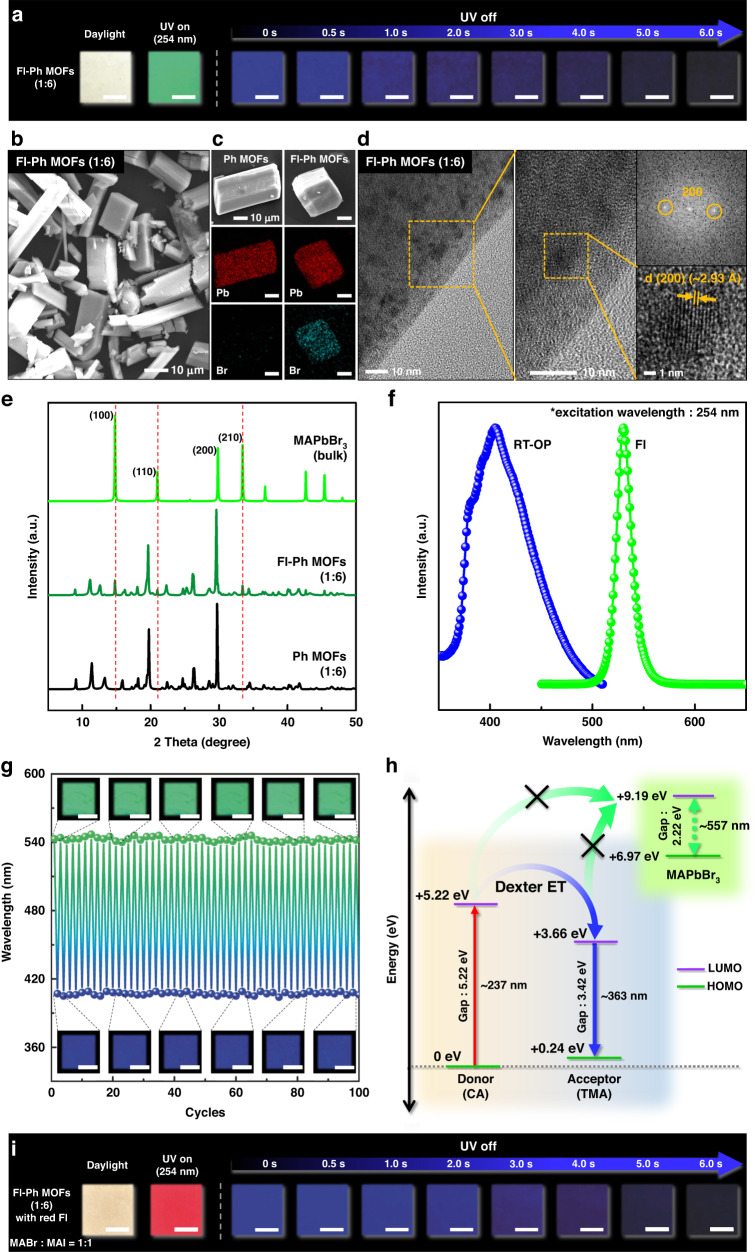
Characterization of MAPbBr3 and MAPbBrxI3-x perovskite NC-embedded Fl–Ph MOFs. **a** Photographs of the Fl–Ph MOF under daylight, UV light (254 nm), and after turning off the UV lamp (scale bars: 1 cm). **b** SEM image of the Fl–Ph MOF. **c** SEM–EDX elemental mapping images of the Ph MOF and Fl–Ph MOF (Pb and Br mapping). **d** HR-TEM images and FFT pattern from HR-TEM image of Fl–Ph MOF. **e** XRD patterns of the Ph MOF, MAPbBr_3_ (bulk), and Fl–Ph MOF. **f** PL spectra of the Fl–Ph MOF under 254 nm excitation. **g** Reversible PL and RT-OP switching test with the Fl–Ph MOF (scale bars: 5 mm). **h** Proposed mechanism of the dual light emission of the Fl–Ph MOF using relative frontier orbital energies. **i** Photographs of the red fluorescent Fl–Ph MOF with iodide containing perovskite (MAPbBr_x_I_3-x_) NCs under daylight, UV light (254 nm), and after turning off the UV lamp

The synthesized Pb-MOF with Pb ions in its molecular framework enabled convenient MOF-template synthesis of Pb-based fluorescent perovskite NCs, i.e., MAPbBr_3_ NCs^35^. Particularly, we synthesized fluorescent MAPbBr_3_ NCs using the Ph MOF (1:6) template to develop a novel dual-light-emitting MOF with both fluorescence and phosphorescence emissions. MAPbBr_3_ NCs were fabricated in the Ph MOF (1:6) by adding an MABr/methanol solution to a dispersion containing the Ph MOF in hexane with vigorous stirring at room temperature, followed by washing and drying^35^. The detailed MAPbBr_3_ synthesis procedure is described in the Experimental Section. We denoted the perovskite NC-embedded MOFs as Fl–Ph MOFs because they exhibit both fluorescence and phosphorescence (Fig. 3a). The Fl–Ph MOF (1:6) sample was successfully produced as a characteristic triclinic powder, and its dimensions were similar to those of the Pb-MOF and Ph MOF samples (see Fig. 3b). The presence of MAPbBr_3_ NCs in the Fl–Ph MOF powder was further confirmed via SEM–energy dispersive X-ray (EDX) Br mapping (Fig. 3c)^31,35^. Furthermore, HR-TEM images show that MAPbBr_3_ NCs are embedded inside Fl–Ph MOF (Fig. 3d)^35^. From the fast Fourier transformation (FFT) images (Figs. 3d, S7), the interplanar distances of approximately 2.93 Å, corresponding to the (200) crystal faces of cubic MAPbBr_3_, were clearly confirmed^36^. The characteristic X-ray energy corresponding to Br atoms was observed in the EDX profile of the Fl–Ph MOF, whereas the same signal was rarely detected for the Ph MOF. The crystalline structure of the perovskite NCs grown on the Ph MOF template was examined via XRD, and the pattern in Fig. 3e clearly presents three new distinct peaks at 14.9°, 21.2°, and 33.7°, corresponding to the (100), (110), and (210) planes of cubic MAPbBr_3_ (space group: *Pm3m*; No. 211)^37,38^.

The characteristic deep-blue RT-OP emission of the Fl–Ph MOF was also visualized as an afterglow after the UV lamp was turned off, as presented in the series of photographs in Fig. 3a. Owing to a large difference in intensity between the fluorescence and RT-OP emissions, the transient blue RT-OP was concealed in the green fluorescence during UV exposure (Fig. 3a). The Fl–Ph MOF emitted green fluorescence with an emission maximum of 530 nm and a quantum yield of 31.44% under 365 nm excitation (Fig. 3f)^35^. The dual light emission of the Fl–Ph MOF upon UV exposure was stable after repetitive switching on/off cycles of the UV lamp over 100 cycles, as presented in Fig. 3g. The Fl–Ph MOF also maintained its initial Fl and RT-OP intensities over 3 days upon exposure to an atmosphere with 10% RH (Fig. S8). In addition, bulk MAPbBr_3_ perovskite powder was synthesized under the same conditions for comparison^39^. Due to the poor stability of pure MAPbBr_3_ crystal powder in air^37^, the initial fluorescence intensity was rapidly dropped when exposed to an atmosphere with a relative humidity of 10%. After 12 h exposure, only 10 % of the initial intensity remained, as shown in Fig. S9. The superior air stability of Fl–Ph MOFs was attributed to the protective effect of the MOF matrix^35^. The fluorescence at the wavelength of 530 nm disappeared instantaneously when the UV lamp was turned off, whereas the RT-OP at the wavelength of 405 nm was clearly visible. After a few seconds, the RT-OP emission diminished in intensity.

First-principles density functional theory (DFT) calculations were used to elucidate the mechanism underlying the dual light emission of the Fl–Ph MOF. For this, first, we modeled the three primary optoelectronic components of the Fl–Ph MOF, that is, CA, TMA, and MAPbBr_3,_ using separate non-interacting models. As depicted in Fig. 3h, by using the relative frontier orbital energy levels, the conduction band minimum (CBM) and valence band maximum of MAPbBr_3_ were found to be significantly elevated relative to the lowest unoccupied molecular orbital levels of CA and TMA. This significant elevation of the CBM of MAPbBr_3_ indicates that dexter energy transfer is not feasible between the perovskite NC and donor/acceptor materials. We also examined possible facilitated electronic transitions between the donor/acceptor and perovskite due to close proximity between the different components. The results (Fig. S10) show that the addition of TMA resulted in the generation of mid-gap states with lower energy levels compared to the perovskite conduction states. This result indicates that spontaneous electronic transitions are not possible between TMA with lower energy levels and the perovskite CBM with higher energy levels, even when TMA and perovskite are in close proximity. In contrast, CA has electronic states that overlap with those of MAPbBr_3_ above the Fermi level. However, electronic transitions between these two materials are rarely feasible owing to spatial separation between the CA and MAPbBr_3_ clusters in the MOF (Fig. S10d). Furthermore, we developed Fl–Ph MOFs with the independent red fluorescence and blue phosphorescence emission, based on MAPbBr_x_I_3-x_ NCs by adding certain amount of methylammonium iodide (MAI) in the perovskite precursor solution (Fig. 3i).

### Dual-light-emitting 2D encryption with Fl–Ph MOFs: pochoir patterning

The synthesized Fl–Ph MOFs are advantageous for optical information encryption and anti-counterfeiting for the following reasons: First, the synthesized MOF powder with excellent solvent resistance can be used to prepare an ink that is suitable for a variety of solution-processible coating technologies such as bar-, screen-, and spin-coating ones. Such solution-processible Fl–Ph MOFs can be readily printed and patterned based on appropriate processes. This capability allows the 2D patterning and printing of information (*vide infra*). In addition, the synthesized Fl–Ph MOF can be dispersed in a polymer solution, and the mixture can be casted to generate an emissive polymer composite. A moldable Fl–Ph MOF-based polymer composite can be employed in conventional 3D-printing technologies, as will be demonstrated later. Moreover, owing to the dual-light-emission characteristics of our Fl–Ph MOF at two distinctive wavelengths of 405 and 530 nm, two different types of optical information with different wavelengths can be written and read as desired. In addition, the decay behaviors of the fluorescence and RT-OP emissions with substantially different decay times of a few nanoseconds and seconds, respectively, may be useful for information encryption. Overall, two types of optical information written based on fluorescence and RT-OP can be independently read on different time scales. The developed 2D- and 3D-patternable Fl–Ph MOFs with dual-light-emitting capability allowed us to develop novel 2D and 3D encryption technologies.

**Fig. 4:**
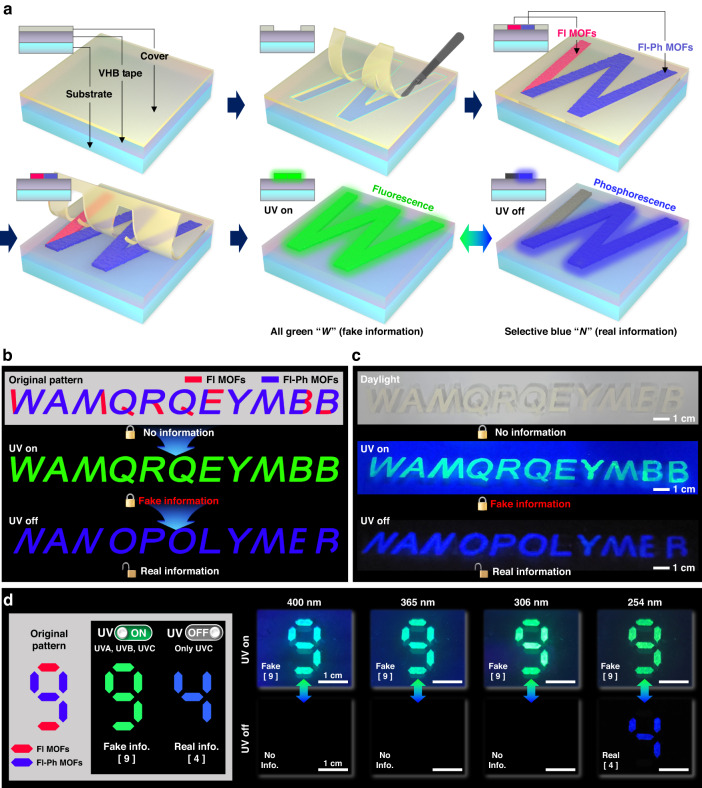
Pochoir patterning of the Fl MOF and Fl–Ph MOF powders for 2D information encryption. **a** Schematic illustration of 2D pochoir patterning with the Fl MOF and Fl–Ph MOF powders. **b** Original 2D pochoir pattern design, and **c** photographs of real information with blue phosphorescence (*NANOPOLYMER*) concealed in fake information with green fluorescence (*WAMQRQEYMBB*) under UV (254 nm) exposure. **d** Photographs of 2D pochoir information encryption and decryption with different excitation wavelength

We developed a dual-light-emitting 2D encryption technique based on pochoir patterning using the synthesized Fl–Ph MOF. For efficient encryption, we used two different MOFs: Fl MOFs and Fl–Ph MOFs. As depicted in Fig. 4a, the MOFs were directly printed on the surface of VHB tape (3 M) using an art technique called pochoir. The following meaningless letters “*WAMQRQEYMBB*” were printed using a combination of the Fl–Ph and Fl MOFs. Specifically, to encrypt “*NANOPOLYMER*,” some parts of “*WAMQRQEYMBB*” were printed using the Fl MOF, whereas the other parts were written using the Fl–Ph MOF. For example, the left-hand side of character “*W*” was printed using the Fl MOF, whereas the rest of it was printed using the Fl–Ph MOF, as presented in the scheme of Fig. 4a. In “*WAMQRQEYMBB*,” the red-colored regions were printed using the Fl MOF and the blue-colored regions using the Fl–Ph MOF, as presented in the top scheme of Fig. 4b. When the printed pattern was exposed to UV light, the letters “*WAMQRQEYMBB*” appeared with bright green fluorescence. However, when the UV lamp was turned off, the fluorescent regions patterned using the Fl MOF disappeared immediately. In contrast, the regions patterned using the Fl–Ph MOF retained their encrypted information, exhibiting their characteristic blue phosphorescence, as shown schematically in Fig. 4b.

Under daylight, the pochoir-patterned letters “*WAMQRQEYMBB*” printed using two types of white MOF powders were barely visible when written on a white substrate, as illustrated in the top photograph of Fig. 4c. As expected, the letters became apparent with bright green fluorescence when the pattern was irradiated with 254 nm UV light (the middle photograph of Fig. 4c). The encrypted information in the form of “*NANOPOLYMER*” was clearly visible with blue RT-OP emitted from regions patterned using the Fl–Ph MOF when the UV light was turned off (Fig. 4c). Note that the actual information encrypted using the Fl–Ph MOF became visible only after the UV lamp (254 nm) was turned off. When the pochoir pattern was exposed to UV light with higher wavelengths, such as 306, 365, and 400 nm, only the fake pattern was apparent with the characteristic green fluorescence, and no real information appeared even after the UV light was turned off (Fig. 4d). Thus, the developed write-once-read-many-times (WORM)-type encryption using Fl and Fl–Ph MOFs combined with the conventional pochoir printing was reliable without significant degradation of the RT-OP emission following multiple reading processes (Supplementary Video 2)^9,11^.

### Dual-light-emitting 3D encryption with Fl–Ph MOF-containing polymer composites: 3D printing

**Fig. 5:**
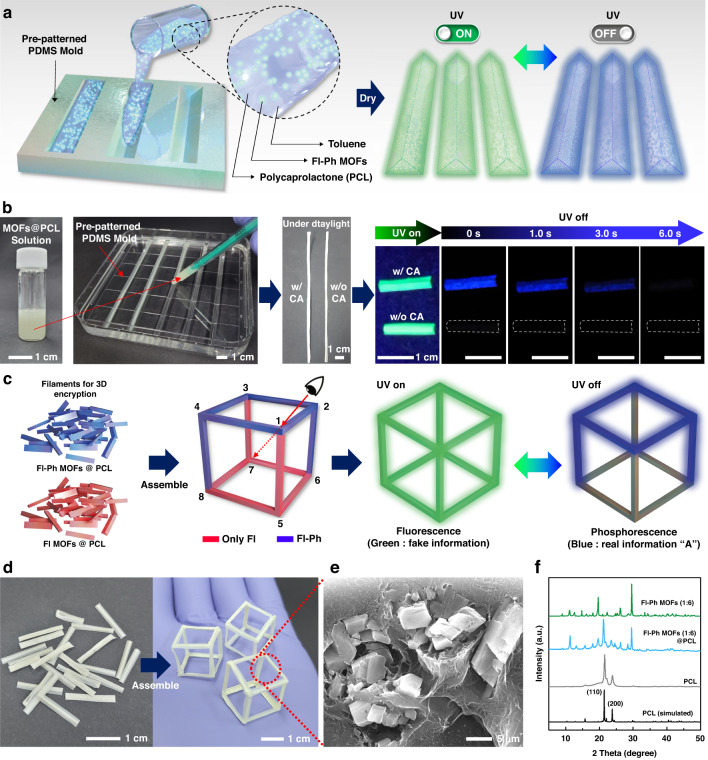
Fabrication of Fl–Ph MOF-containing PCL polymer filaments for 3D encryption with a smartphone. **a** Schematic illustration of Fl MOF and Fl–Ph MOF particle-embedded PCL filament fabrication using a pre-patterned PDMS mold. **b** Photographs of the Fl–Ph MOF@PCL filament fabrication process with maintained dual light emission. **c** Schematic illustration of 3D-encrypted cube construction, with real information with blue phosphorescence concealed in the fake information with green photoluminescence under UV exposure. **d** Photographs of the Fl–Ph MOF@PCL filament and assembled 3D cubes. **e** Cross-sectional SEM image and **f** XRD patterns of the Fl–Ph MOF@PCL filaments

**Fig. 6:**
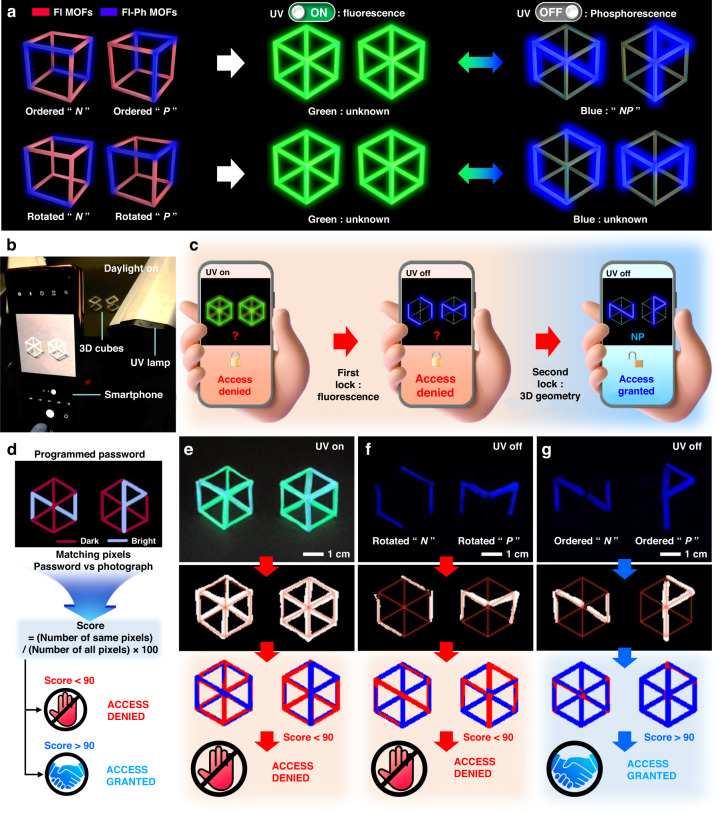
3D double encryption with an Fl–Ph MOF-dispersed 3D-printable PCL polymer matrix for smartphone application. **a** 3D double encryption of a cube structure with Fl–Ph MOF@PCL filaments. **b** Operation of the smartphone application for 3D double encryption using a conventional camera. **c** Process of reading the encrypted information with blue phosphorescence hidden under fluorescence using a smartphone application. **d** Reading the results of 3D-encrypted information by **e** using dual-light-emitting cubes under UV (254 nm) light, **f** using rotated cubes after the UV lamp was turned off, and **g** using correctly aligned cubes after the UV lamp was turned off for the encryption of “*NP*” (top: photograph captured by smartphone, middle: binarized image, bottom: password comparison result with scores)

We successfully employed our Fl–Ph MOF for novel pattern encryption relying on pattern recognition from a viewing-angle-dependent 2D projection of a 3D-architectured structure using Fl–Ph MOF/polymer composites (see Figs. 5, 6). For this, first, the filament components of a 3D skeletal structure were fabricated using polymer composites comprising polycaprolactone (PCL) and the Fl MOF or Fl–Ph MOF. The filament components were fabricated by casting a solution of PCL in toluene dispersed with the MOF powder in a pre-patterned polydimethylsiloxane (PDMS) mold. The casted solution was dried under vacuum at room temperature, as schematically presented in Fig. 5a. Two types of PCL filaments were prepared: one with the Fl–Ph MOF (denoted as Fl–Ph MOF@PCL), and another with the Fl MOF (denoted as Fl MOF@PCL), as depicted in Fig. 5b. The fabrication process is detailed in the Experimental Section. We also confirmed that the Fl–Ph MOF@PCL filament presented both fluorescence and RT-OP emissions, whereas the Fl MOF@PCL filament only presented fluorescence under UV light, without RT-OP (Fig. 5b). Furthermore, we confirmed that various polymers, such as polystyrene, polymethylmethacrylate, and ethyl cellulose, could be used to prepare filaments containing the Fl–Ph MOF (Fig. S11). By employing the two types of MOF composite filaments, we developed a regular hexahedral skeleton, as schematically illustrated in Fig. 5c.

Notably, encryption can be accomplished by viewing a cube along its eight different vertexes, as illustrated in Fig. 5c. Additionally, six different rotational hexagonal projections from each of the eight vertexes can be obtained when the cube is rotated by 60°, implying that 48 different hexagonal projections can be used for encoding and decoding, as illustrated in Fig. S12. A structurally programmed cube composed of the Fl–Ph MOF@PCL and Fl MOF@PCL filaments was fabricated for information encoding based on 48 different hexagonal projections. As illustrated in Fig. 5c, for example, when a cube constructed using the two types of filaments to encode the alphabet “*A*” was viewed along vertex number 1, a hexagon was projected. Under UV exposure, a hexagonal fluorescence image appeared. When the UV light was turned off, the encoded alphabet “*A*”, as illustrated in Fig. S13, appeared with blue RT-OP emission from six programmed Fl–Ph MOF@PCL filaments, as depicted in Fig. 5c. The other 47 hexagons projected from the other vertexes with a rotational symmetry of six also presented the same hexagonal fluorescence image under UV exposure. However, the phosphorescent images obtained after turning off the UV lamp were all different from the encoded information “*A*,” facilitating a novel octonary encoding platform. Again, the encoded information from the Fl–Ph MOF filaments was only shown after UV lamp (254 nm) was turned off.

In addition, two possibilities of true and false for each of the 12 edges of a cube should be considered for cube counterfeiting. Therefore, a total number of 2^12^ × 48 (= 196,608) cases should be considered to counterfeit one cube, which mandates a much longer cracking time than that required when cracking a conventional character password system (Table S2). Upon adding *n* more cubes to the platform, (2^12^ × 48)^*n*^ combinations are possible for information encoding. Thus, information security can be exponentially enhanced by adding more cubes. For example, if a password can be cracked at a rate of one million times per second in a system, it would only take less than a second to crack a password programmed with three words of alphanumeric characters. On the other hand, cracking a password encoded with three cubes would take 241 years (Table S2). Furthermore, by designing a 2D-projected pattern from a cube containing two types of composites, a variety of information, such as digits, drag lock patterns, and paths, could be encrypted. This versatility illustrates the significant potential of our dual-light-emitting filaments in information encryption. To demonstrate the suitability of 3D encryption based on our dual-light-emitting filaments, a cube with an edge length of 1.5 cm was constructed with 12 filaments of Fl–Ph MOF@PCL and Fl MOF@PCL, as presented in Fig. 5d. The cross-sectional SEM image of Fl–Ph MOF@PCL reveals that the MOF powder particles were securely embedded in the PCL matrix, indicating no decomposition or degradation of the MOF particles during the filament fabrication process (Fig. 5e). The structural stability of the dispersed MOF particles was also confirmed via XRD, which also indicated excellent preservation of the crystal structure and crystallinity (Fig. 5f)^35^.

### 3D Double encryption with a smartphone camera

Two cubes with different combinations of the Fl–Ph MOF@PCL and Fl MOF@PCL filaments were designed, and these were intended to present (password) “*N*” and “*P*” when projected appropriately from their vertexes. The password “*NP*” could be observed in the RT-OP mode when both cubes were appropriately viewed from the given vertexes, as schematically illustrated in Fig. 6a (also see Supplementary Video 3). A facile decoding process allowing the verification of the password “*NP*” was developed by capturing an RT-OP pattern from the two cubes using a conventional smartphone camera. This process was followed by image processing with a custom-built smartphone application, as depicted in Fig. 6b and Supplementary Video 4. The decoding process using the two cubes was employed to unlock a conventional smartphone, as schematically presented in Fig. 6c. When the two cubes were exposed to UV light, the characteristic hexagonal patterns of the two cubes could be observed owing to the associated strong fluorescence. Immediately after the distinction of the hexagonal patterns, the flashlight was turned off to observe the RT-OP patterns. The image of the two RT-OP patterns resulting from the two cubes was captured by the smartphone camera, and this was followed by a pattern comparison with the password “*NP*” using a software to determine whether the smartphone could be unlocked or not.

Password verification was accomplished based on a pixel-matching process developed by us, and this was conducted between a captured RT-OP pattern and a pre-programmed real password pattern, as illustrated in Fig. 6d. The pixel-matching algorithm of the custom-built application is also presented in Fig. S14. In brief, the brightness of all pixels in a captured photograph is classified as zero or one based on the original brightness. Essentially, the original brightness is graded from zero to 255. If the brightness of a certain pixel is greater than 128, it is classified as one; otherwise, it is classified as zero. After this binarization process of the photograph, as presented in Fig. S15, the binarized image is compared with the pre-programmed password (“*NP*”) image. The greater the match between the pixels, the higher the score (0 ~ 100%) awarded. If the score is higher than 90%, access is granted; otherwise, access is denied (Fig. S15). As presented in Fig. 6e–g, access was granted only when the captured pattern “*NP*” with the RT-OP emission was entered as a password. On the other hand, access was denied for all other RT-OP patterns resulting from different combinations of the two cubes, as well as from different vertex viewing directions (Fig. S16). The entire operation of our custom-built 3D encryption process based on the developed dual-light-emitting MOF composites is also demonstrated in Supplementary Video 5. The obtained results indicate that 3D encryption based on simple cubes comprising the synthesized dual-light-emitting MOF/polymer composites can be used as a smartphone locking/unlocking system with high-security levels. In addition, our portable cube can be scaled down, allowing the realization of a miniaturized or micro-scale locking system. By simply increasing the number of cubes, the security level can be exponentially increased (Table S2). Furthermore, the slow self-extinction of the RT-OP patterns may be beneficial because the system does not require additional pattern-erasing steps. The solid-state powder-type MOFs with distinct visible-range dual light emissions based on fluorescence and RT-OP presented in this paper demonstrate considerable potential in encryption and anti-counterfeiting, particularly in applications based on large area, high-throughput printing technologies, and can inspire the design of novel MOF-based multi-light-emitting materials.

## Discussion

Herein, we demonstrated high-security and fidelity solid-state optical encryption based on fluorescent and phosphorescent dual-light-emitting MOFs in combination with 2D- and 3D-printing technologies. Solid-state RT-OP emissions were achieved by rigidifying the MOF containing host molecules of TMA with CA as the guest molecules. Deep-blue (405 nm) RT-OP appeared reliably, regardless of the RH condition, even when the phosphorescent MOF was immersed in various organic solvents. By utilizing the metal ion (Pb^2+^) of the phosphorescent MOF, fluorescent MAPbBr_3_ and MAPbBr_x_I_3-x_ NCs were synthesized to produce a novel dual-emission MOF, which presented intense green and red fluorescence under UV exposure, respectively, in addition to its characteristic blue phosphorescence. Our solid-state dual-light-emitting MOFs were successfully employed in 2D pochoir patterning, realizing printable WORM 2D encryption. More importantly, a high-security 3D-printable optical encoding/decoding system was demonstrated with moldable polymer composites of the dual-light-emitting MOFs. Viewing-angle-dependent encryption based on cube-type skeletons composed of the polymer composite filaments was successfully accomplished with a conventional smartphone camera and custom-built software. Thus, this study not only provides a novel design strategy for developing solid-state multiwavelength light-emitting materials suitable for a variety of wearable, patchable, and stretchable sensors and displays but also presents a high-security information encryption technique suitable for personal biodata protection technologies, which are rapidly progressing owing to human-machine interface research^40–42^.

## Materials and methods

### Materials

Lead nitrate (Pb(NO_3_)_2_, 99.0%), TMA (95%), CA (98%), and other solvents, including hexane (anhydrous, 99.9%), benzene (anhydrous, 99.9%), toluene (anhydrous, 99.9%), chlorobenzene (anhydrous, 99.9%), chloroform (anhydrous, 99.9%), diethyl ether (anhydrous, 99.9%), methanol (anhydrous, 99.9%), ethanol (anhydrous, 99.9%), isopropanol (anhydrous, 99.9%), DMF (anhydrous, 99.9%), and DMSO (anhydrous, 99.9%), were purchased from Sigma–Aldrich and were used as received. CH_3_NH_3_Br (MABr) was purchased from Xi’an Co.

### Preparation of the Pb-MOF and Ph MOFs

The Pb-MOF was synthesized via a conventional solvothermal reaction between Pb and TMA. A solution of TMA (0.6962 g) in 320 mL of DI water was stirred at 63 °C for 1 h to form a clear solution. Following this, a clear solution of Pb(NO_3_)_2_·6H_2_O (4.1802 g) in 30 mL of water was added to the ligand solution, and the mixture was stirred vigorously at 63 °C for 1 h. A white precipitate was formed, which was then washed thrice with DI water and collected through centrifugation. These purification steps were repeated 3-times. The obtained white product was dried under vacuum for 12 h at 70 °C. Further, Ph MOFs (x:1) were synthesized in the same manner using a TMA/CA ligand solution, wherein the CA molar concentration was x times higher compared to the TMA concentration.

### Synthesis of MAPbBr_3_ NCs on the Pb-MOF and Ph MOF

MAPbBr_3_ NCs were synthesized by adding 500 μL of an MABr/methanol solution (1.0 mg mL^−1^) to a suspension of the Pb-MOF or Ph MOFs (200 mg) in 10 mL of hexane. The MAPbBr_3_ NCs templated MOFs were rinsed with 20 ml of hexane and n-butanol, and collected via centrifugation. These purification steps were repeated 3-times. The precipitates were dried under vacuum for 12 h at room temperature to prevent thermal degradation of the perovskite NCs.

### Fabrication of Fl–Ph MOF@PCL filaments using a PDMS mold

A pre-patterned elastomeric PDMS (Sylgard 184) mold was fabricated by curing PDMS with a curing agent at a weight ratio of 10:1 in an oven at 60 °C for 12 h. The completely cured PDMS plate was then engraved with a line pattern using a razor blade. The MOF powder (0.096 g) and PCL polymer pulp (0.2 g) were dispersed in toluene (0.8 g). After the PCL was completely dissolved in toluene, the solution was poured into the engraved line patterns of the PDMS mold and vacuum dried for 3 h at room temperature for toluene evaporation. The Fl–Ph MOF@PCL filament was finally detached from the PDMS mold after the drying process.

### Characterization

The surface morphologies and elemental distributions of the MOF particles were examined using a field emission-SEM instrument at an acceleration voltage of 20.0 kV (IT-500, JEOL, Japan). High-resolution transmission electron microscopy (HR-TEM) images were acquired from JEM-F200 (JEOL, Japan) transmission electron microscope with an accelerating voltage of 200 kV. Powder XRD patterns were recorded using a Rigaku SmartLab instrument. The FT-IR spectra were obtained using Jasco FT/IR-4700 (JASCO Global, Japan). The decomposition temperatures of the samples were determined based on thermogravimetric analysis (TGA1 Mettler TOLED) by heating them from 25 to 900 °C at a rate of 5 °C min^−1^ under air flow (50 cm^3^ min^−1^). The Brunauer–Emmett–Teller pore volume and surface area of each sample were evaluated based on the N_2_ adsorption/desorption isotherm obtained using an Autosorb-iQ 2ST/MP instrument (USA) at 77 K. Before the measurements, each sample was pre-heated at 100 °C under vacuum for 2 h in Autosorb-iQ 2ST/MP. The PL spectra and time-resolved PL decay curves of the powders were obtained using a Fluorolog3 instrument (HORIBA, Japan) equipped with a 254 nm excitation light source at room temperature.

### First-principles calculations

DFT calculations were performed using the projector augmented wave pseudopotential, as implemented in the Vienna Ab initio Simulations Package^43,44^. The plane-wave cutoff energy was set to 400 eV. The electron exchange and correlation were modeled using the generalized gradient approximation based on the Perdew–Burke–Ernzerhof (PBE) functional^45^. For an enhanced description of the van der Waals interactions, DFT-D3 with Becke–Jonson damping was implemented^46,47^. Geometrical optimization of all systems was conducted using the Gamma point. The perovskite surface was modeled by initially optimizing bulk MAPbBr_3_ using a 2 × 2 × 1 supercell structure. Following this, the slab structure was developed by fixing the bottom layers of the perovskite atoms and separating the slabs by a distance of >20 Å to prevent any artificial interaction between them.

### Supplementary information

Supplementary information

Supplementary Video 1

Supplementary Video 2

Supplementary Video 3

Supplementary Video 4

Supplementary Video 5

## Data Availability

The data sets generated and analyzed during this study can be made available from the corresponding author on reasonable request.
