# Supplementary material for: Dual-light emitting 3D encryption with printable fluorescent-phosphorescent metal-organic frameworks
Source: Light Sci Appl. 2023 Sep 12;12:226. doi: 10.1038/s41377-023-01274-4 (PMC10495391; doi:10.1038/s41377-023-01274-4)
Supplement: Supplementary file 1 — Supplementary information [file 41377_2023_1274_MOESM1_ESM.docx]

**Supplementary Information for**

Dual-Light Emitting 3D Encryption with Printable Fluorescent-Phosphorescent Metal-Organic Frameworks

Jin Woo Oh^1^, Seokyeong Lee^1^, Hyowon Han^1^, Omar Allam^2, 3^, Ji Il Choi^3^, Hyeokjung Lee^1^, Wei Jiang^1^, Jihye Jang^1^, Gwanho Kim^1^, Seungsoo Mun^1^, Kyuho Lee^1^, Yeonji Kim^1^, Jong Woong Park^1^, Seonju Lee^1^, Seung Soon Jang^3,^*, and Cheolmin Park^1,4,^*

^1^ Department of Materials Science and Engineering, Yonsei University, Seoul 03722, Republic of Korea

^2^ The George W. Woodruff School of Mechanical Engineering, Georgia Institute of Technology, 801 Ferst Drive, Atlanta, GA 30332-0405, United States

^3^ School of Materials Science and Engineering, Georgia Institute of Technology, 771 Ferst Drive, Atlanta, GA 30332-0245, United States

^4^ Spin Convergence Research Center, Korea Institute of Science and Technology (KIST), Seoul 02792, Republic of Korea

*Corresponding author

Seung Soon Jang

Fax: +404-894-9140 Tel: +404-385-3356 E-mail: seungsoon.jang@mse.gatech.edu

Cheolmin Park

Fax: +82-2-312-5375 Tel: +82-2-2123-2833 E-mail: cmpark@yonsei.ac.kr

**Supplementary Tables 1 and 2**

**Supplementary Figures 1–16**

**Supplementary Movies 1–5**

**Supplementary Video 1.** Stability of the phosphorescence intensity of the Ph MOF (1:6) sample in various solvents.

**Supplementary Video 2.** Movie of real information with blue phosphorescence (*NANO*) concealed in fake information with green fluorescence (*WAMQ*) under UV (254 nm) exposure.

**Supplementary Video 3.** 3D-encrypted cube construction, with real information with blue phosphorescence concealed in the fake information with green photoluminescence under UV exposure.

**Supplementary Video 4.** Reversible Fl and RT-OP switching demonstration with the 3D-encrypted cubes and smartphone.

**Supplementary Video 5.** The entire operation of our custom-built 3D encryption process based on the developed dual-light-emitting 3D-encrypted cubes.


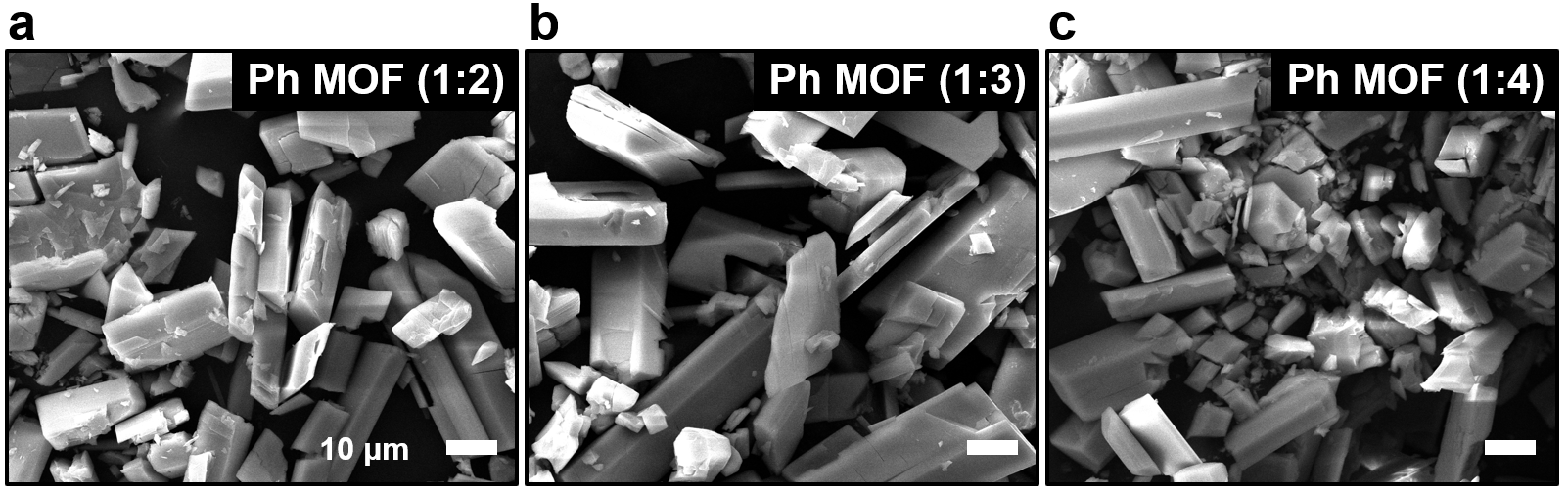


**Supplementary Figure 1.** Scanning electron microscopy images of the as-synthesized (a) Ph MOF (1:2), Ph MOF (1:3), and Ph MOF (1:4) samples. Ph MOF: phosphorescent metal–organic framework.

**
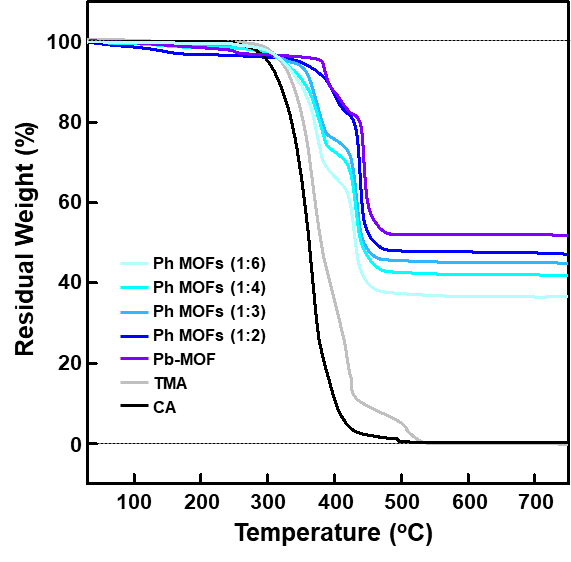
**

**Supplementary Figure 2.** Thermogravimetric analysis curves of cyanuric acid (CA), trimesic acid (TMA), the Pb-MOF, and the Ph MOFs with different CA loadings under air (O_2_/N_2_).

**Supplementary Table 1.** Actual CA loading concentration in the Ph MOFs.

| **Sample** | **Residual**  **(wt%)** | **Pb (wt%)** | **CA**  **(wt%)** | **CA**  **(mol%)** |
| --- | --- | --- | --- | --- |
| CA | 0 | 0 | 100.0 | 100.0 |
| Ph MOF (1:6) | 36.5 | 33.9 | 27.6 | 38.1 |
| Ph MOF (1:4) | 41.8 | 38.8 | 19.3 | 27.9 |
| Ph MOF (1:3) | 44.9 | 41.7 | 13.4 | 20.1 |
| Ph MOF (1:2) | 47.2 | 43.8 | 8.9 | 13.6 |
| Pb-MOF | 51.8 | 48.1 | 0 | 0 |

**
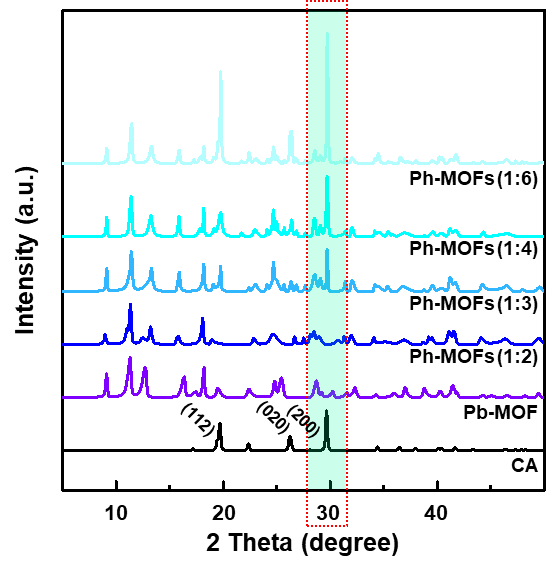
**

**Supplementary Figure 3.** Normalized X-ray diffraction (XRD) patterns of CA, the Pb-MOF, and the Ph MOFs with different CA loadings.


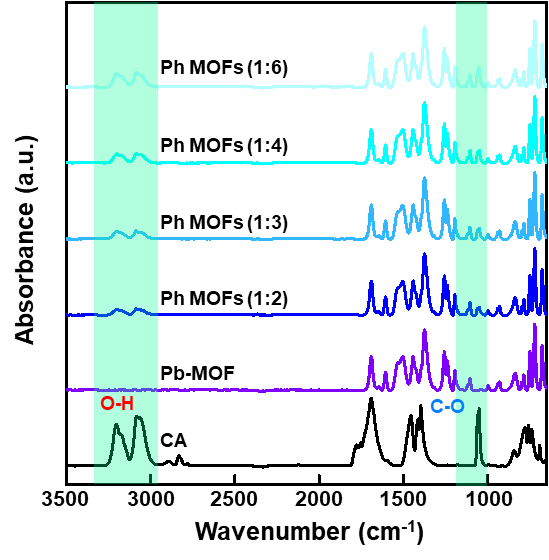


**Supplementary Figure 4.** Fourier transform infrared spectra of CA, the Pb-MOF, and the Ph MOFs with different CA loadings.

**
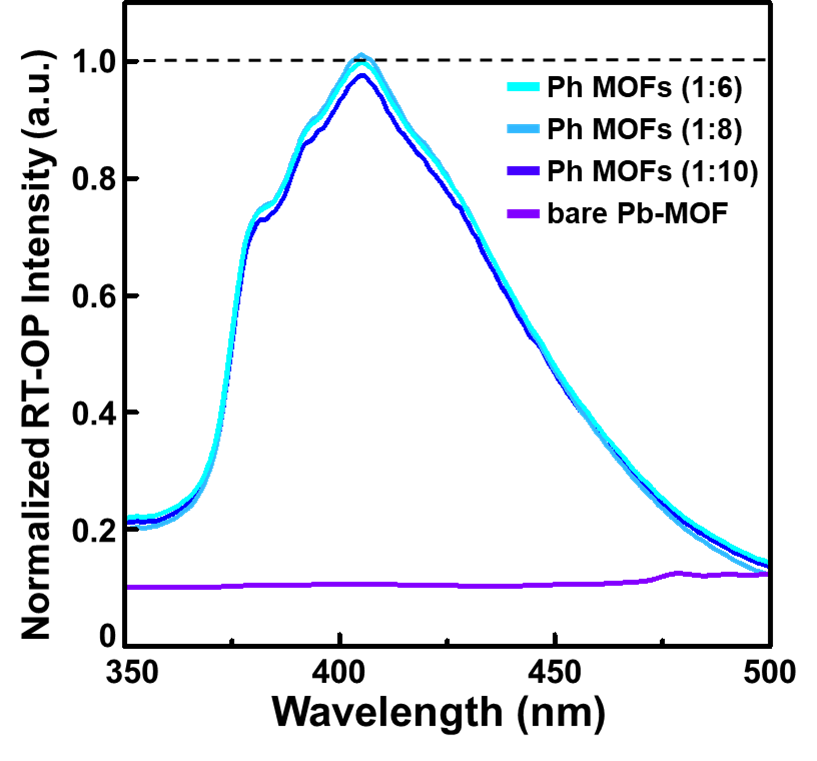
**

**Supplementary Figure 5.** Steady-state RT-OP spectra of the 405 nm emission for the Pb-MOF and Ph MOFs with different CA loadings.


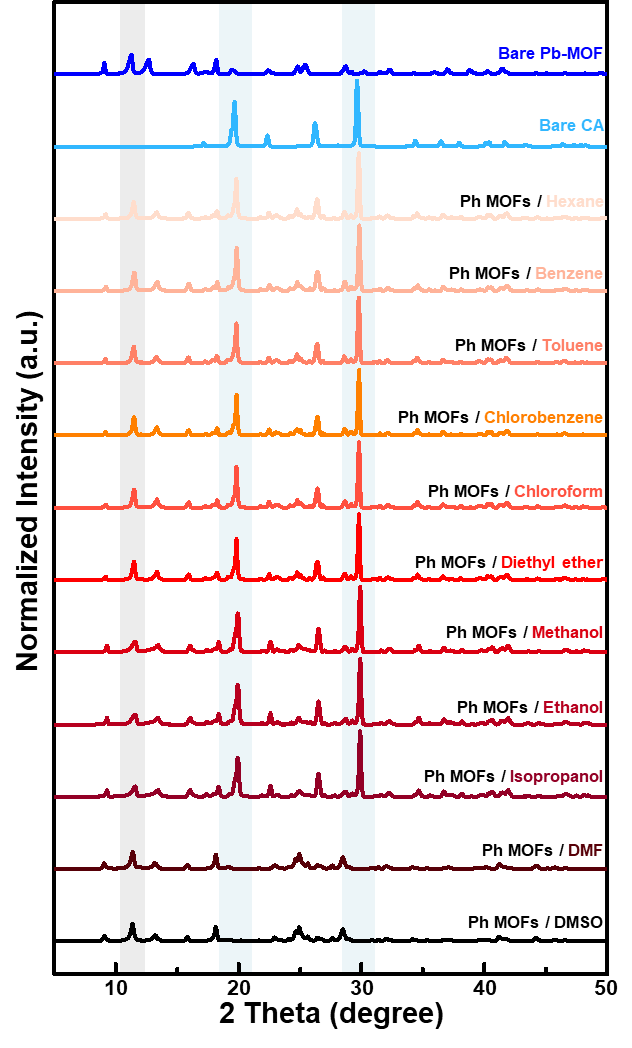


**Supplementary Figure 6.** Normalized powder XRD patterns of the Ph MOF (1:6) particles after treatment with different solvents. The characteristic XRD peaks of the initial Ph MOF (1:6) sample were preserved after it was immersed in poor solvents for CA. However, the Ph MOF (1:6) powder immersed in DMF and DMSO presented a loss of XRD peaks attributed to CA; here, only characteristic reflections originating from the Pb-MOF were retained, confirming the release of CA from the sample.

^
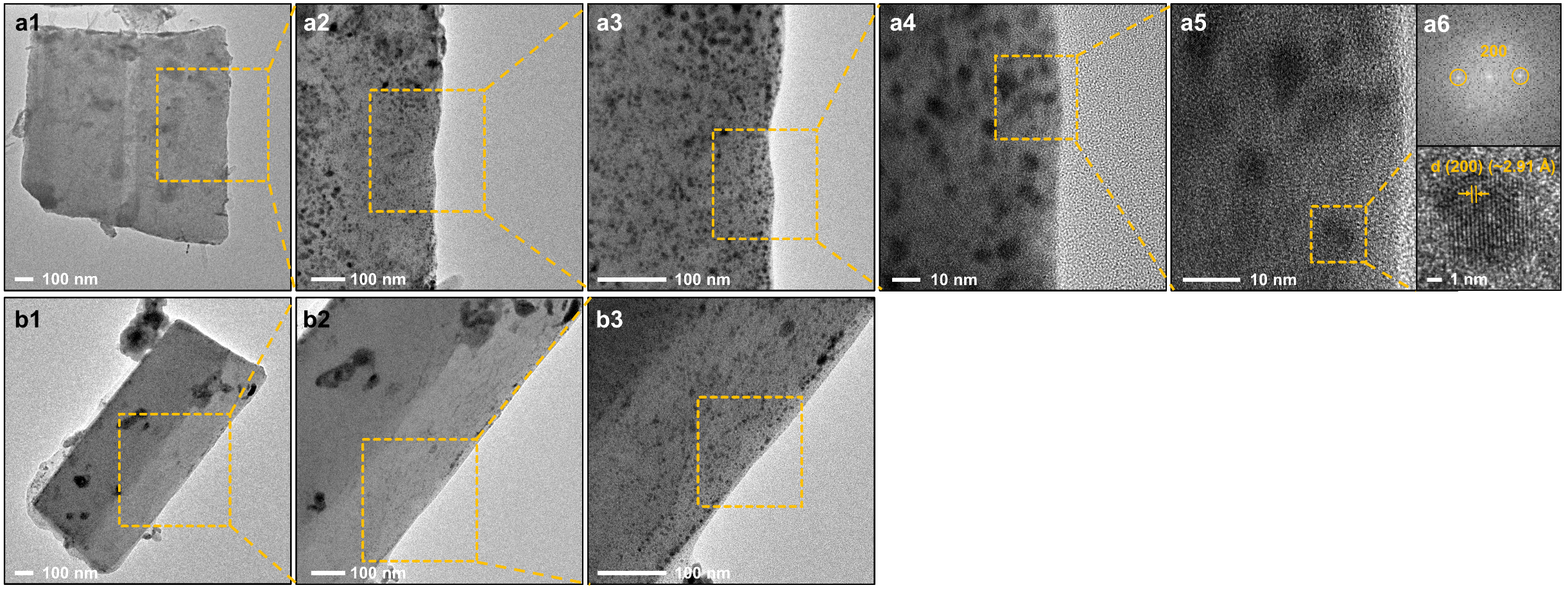
^

**Supplementary Figure 7.** TEM images of (a1-a5) MAPbBr_3_ perovskite templated Pb-MOF and (a6) FFT pattern from HR-TEM image of (a6) MAPbBr_3_ perovskite templated Pb-MOF. (b1-b3) Low-magnification TEM images of Fl–Ph MOF from Fig. 3d.


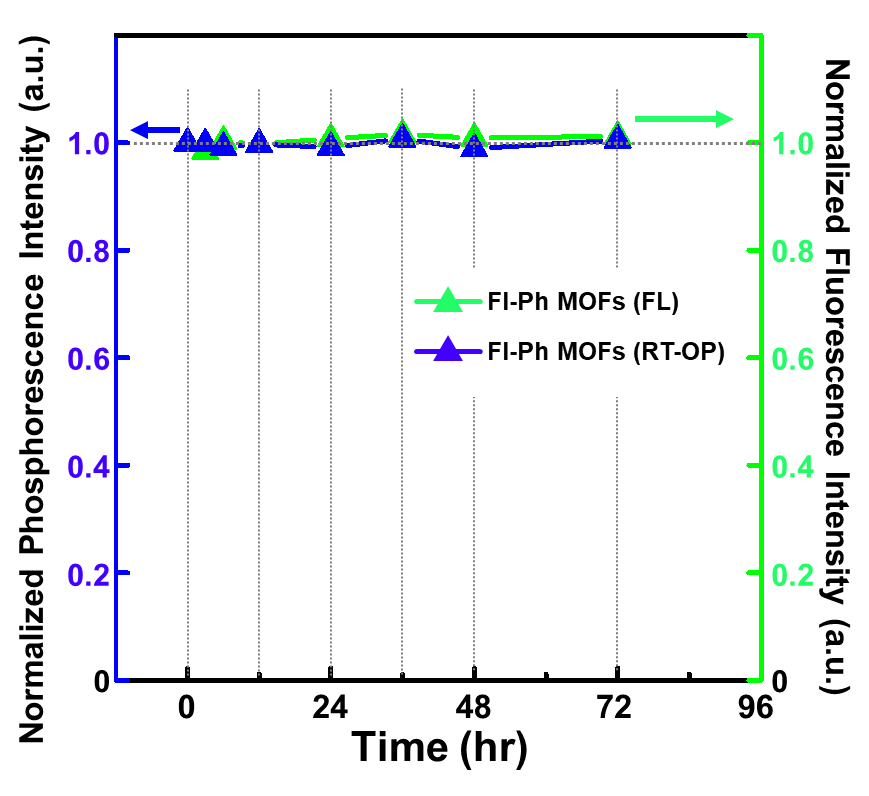


**Supplementary Figure 8.** Phosphorescence and fluorescence intensities of the Fl–Ph MOF exposed to an atmosphere with an RH of 10 % over time.


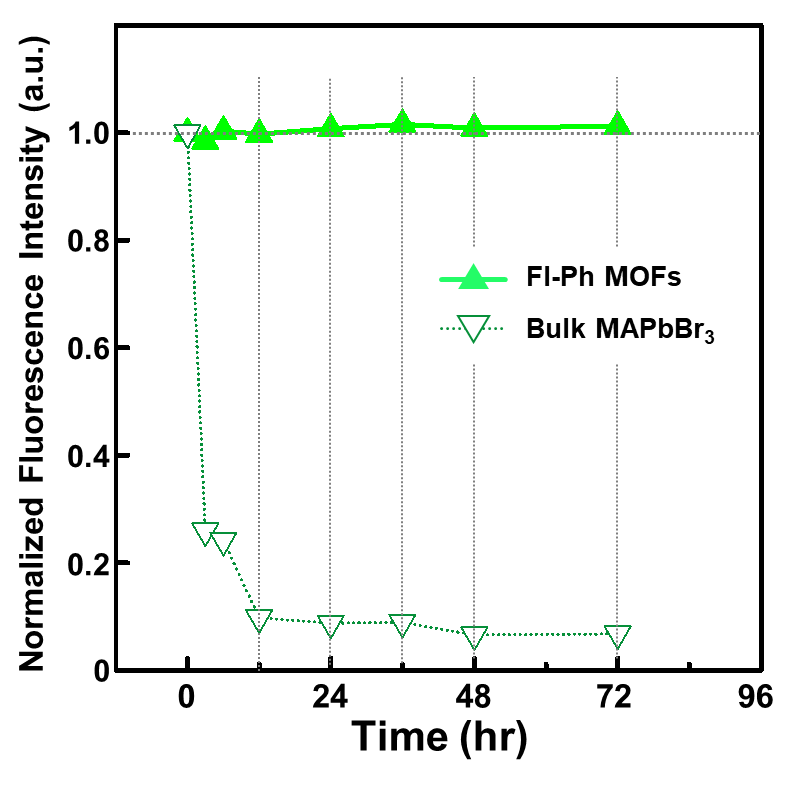


**Supplementary Figure 9.** Fluorescence intensity of the Fl–Ph MOF and bulk MAPbBr_3_ perovskite powder exposed to an atmosphere with an RH of 10 % over time.


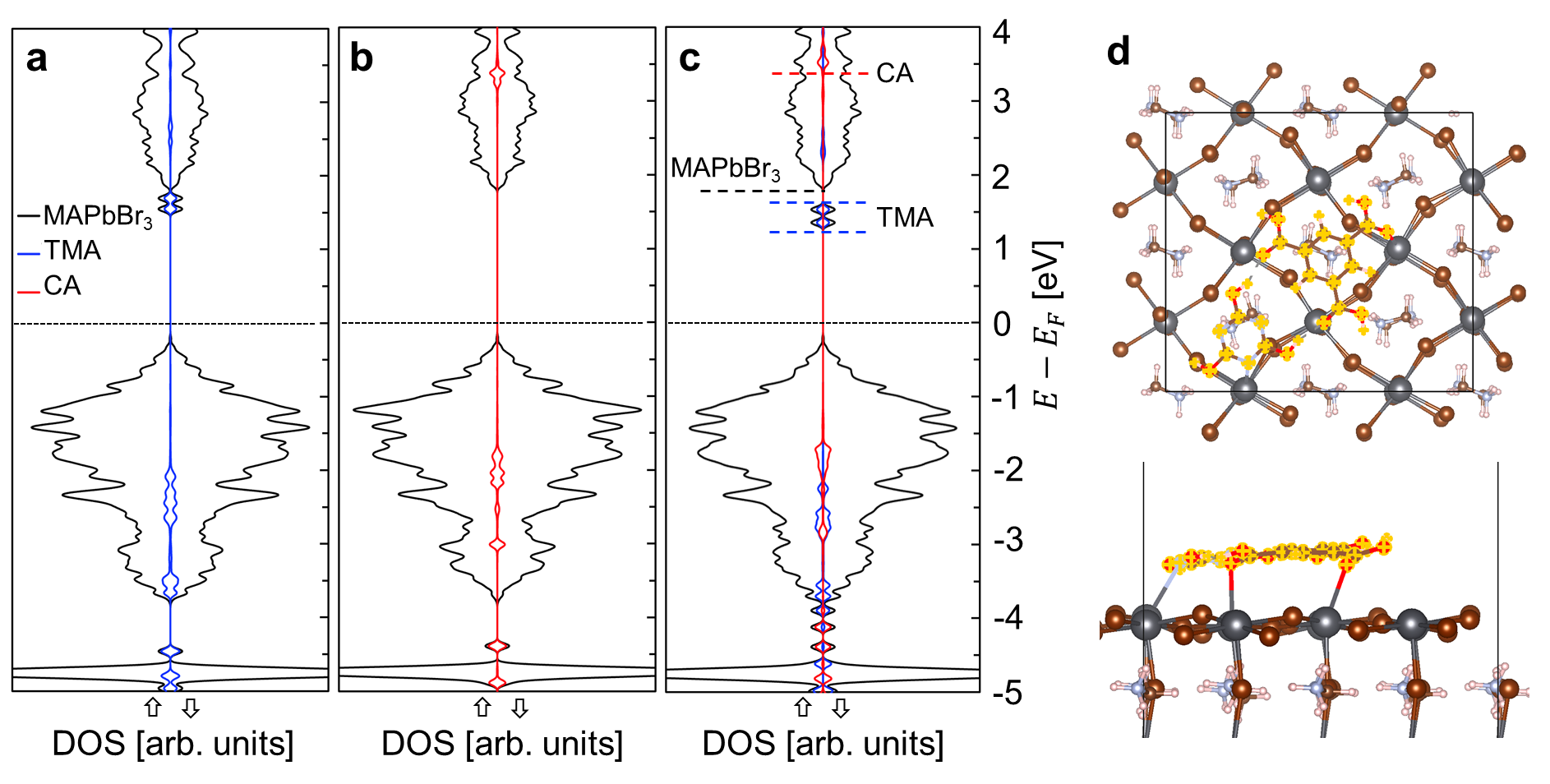


**Supplementary Figure 10.** Density of states for the perovskite surface interacting with (a) TMA, (b) CA, and (c) TMA/CA. (d) Structure of the perovskite surface with TMA and CA.


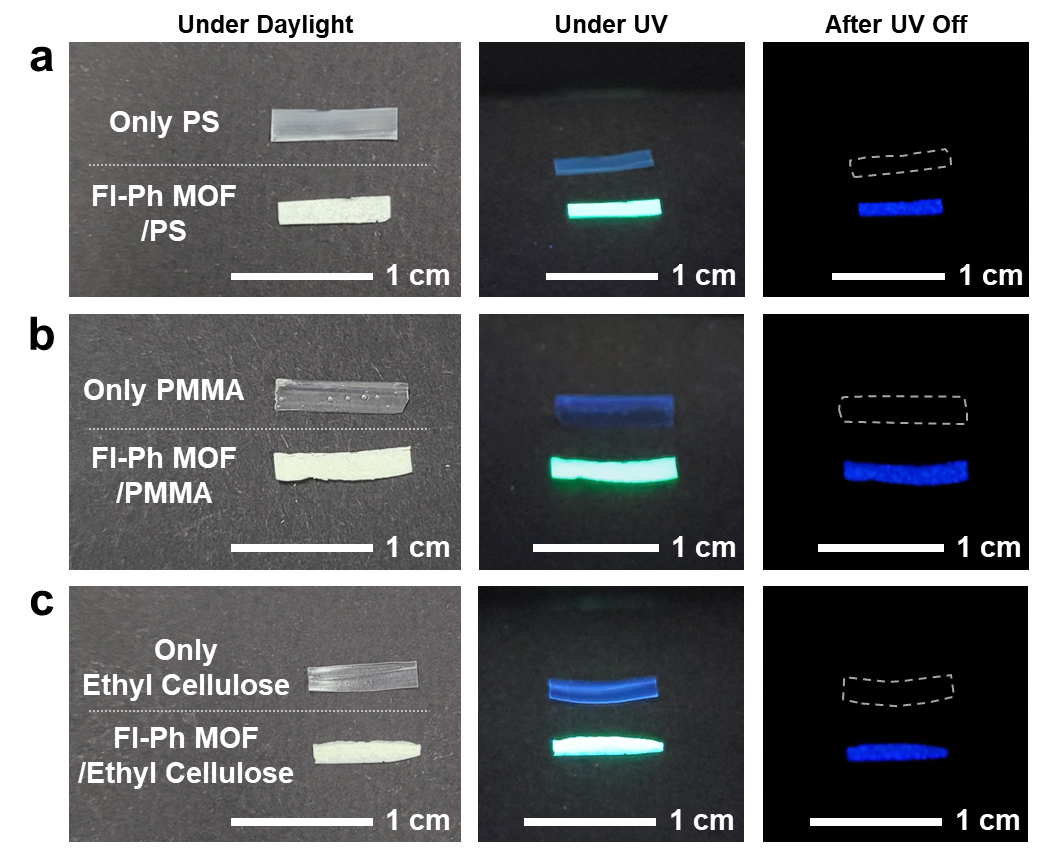


**Supplementary Figure 11.** Photographs of the Fl–Ph MOF@polymer filaments fabricated with (a) polystyrene (280 k), (b) polymethylmethacrylate (120 k), and ethyl cellulose (48 % ethoxyl) as filament matrices.


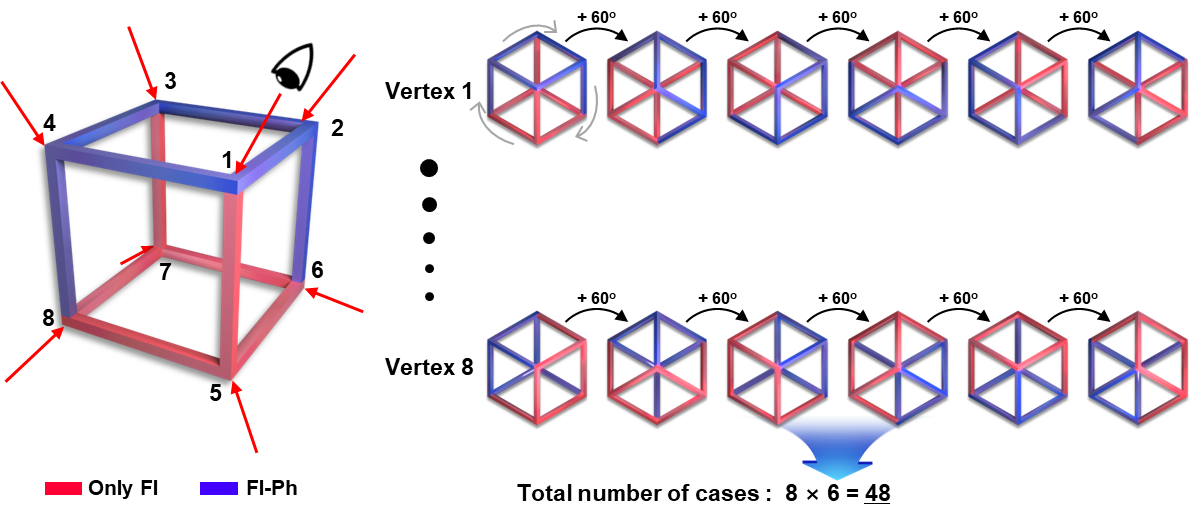


**Supplementary Figure 12.** Total number of cases for hexagonal projection developed with a rotation angle of 60 ° from every eight vertexes of one cube.

**Supplementary Table 2.** Number of combinations and cracking time for different encryption systems.

|  | **Password length (characters or cubes)** | **Alphanumeric characters*** | **All printable characters**** | **Dual-light-emitting**  **3D cube** |
| --- | --- | --- | --- | --- |
| **Number of combinations** | 1 | 62 | 95 | 196,608 |
|  | 2 | 3.84 × 10^3^ | 9.03 × 10^3^ | 9.66 × 10^9^ |
|  | 3 | 2.38 × 10^5^ | 8.57 × 10^5^ | 9.50 × 10^14^ |
|  | 4 | 1.48 × 10^7^ | 8.15 × 10^7^ | 9.34 × 10^19^ |
|  | 5 | 9.16 × 10^8^ | 7.74 × 10^9^ | 9.18 × 10^24^ |
| **Cracking time for one million trials per second** | 1 | 0.062 ms | 0.095 ms | 0.20 s |
|  | 2 | 3.8 ms | 9.0 ms | 10.74 h |
|  | 3 | 0.24 s | 0.86 s | 241 yr |
|  | 4 | 14.78 s | 81.45 s | 4.7 × 10^7^ yr |
|  | 5 | 15.3 min | 129.0 min | 9.3 × 10^12^ yr |

**Alphanumeric characters* :** 0, 1, 2, 3, 4, 5, 6, 7, 8, 9, a, b, c, d, e, f, g, h, i, j, k, l, m, n, o, p, q, r, s, t, u, v, w, x, y, z, A, B, C, D, E, F, G, H, I, J, K, L, M, N, O, P, Q, R, S, T, U, V, W, X, Y, Z

**All printable characters**** : 0, 1, 2, 3, 4, 5, 6, 7, 8, 9, a, b, c, d, e, f, g, h, i, j, k, l, m, n, o, p, q, r, s, t, u, v, w, x, y, z, A, B, C, D, E, F, G, H, I, J, K, L, M, N, O, P, Q, R, S, T, U, V, W, X, Y, Z, , !, “, #, $, %, &, ’, (, ), *, +, ,, -, ., /, :, ;, <, =, >, ?, @, [, ], \, ^, _, `, {, }, ~, |


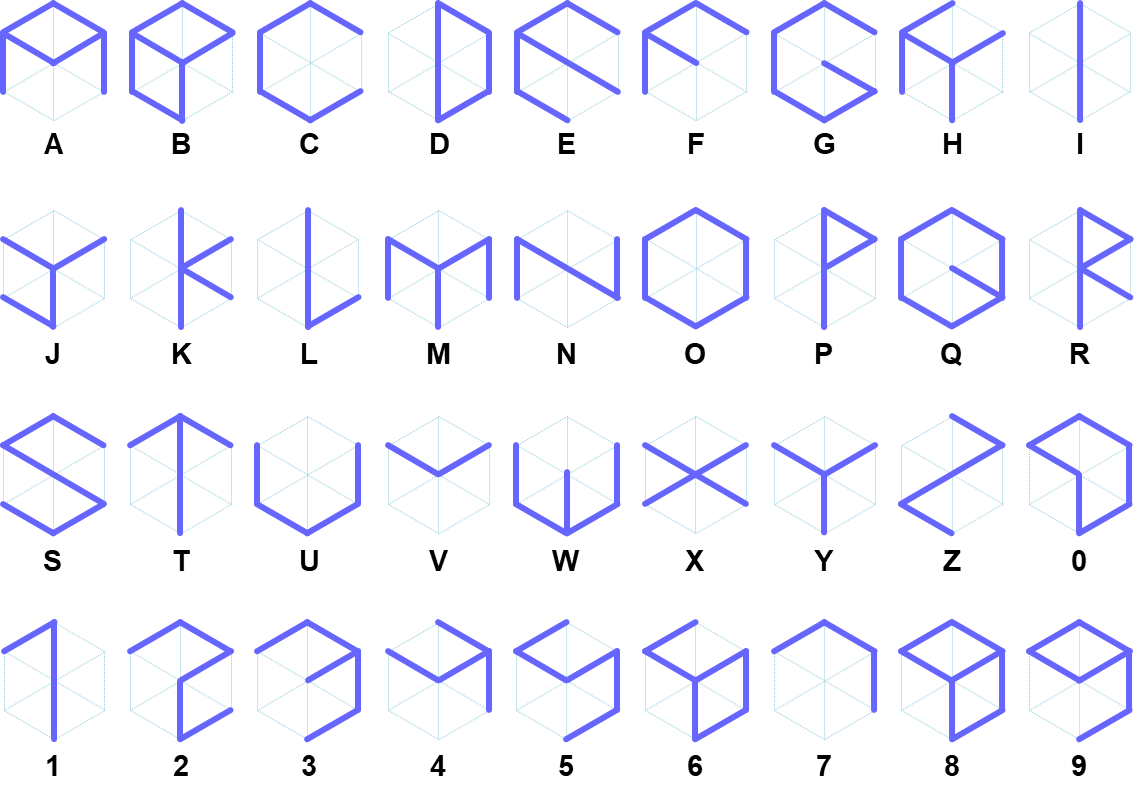


**Supplementary Figure 13.** Examples of projected hexagons for encryption of alphabets and Arabic numerals information.


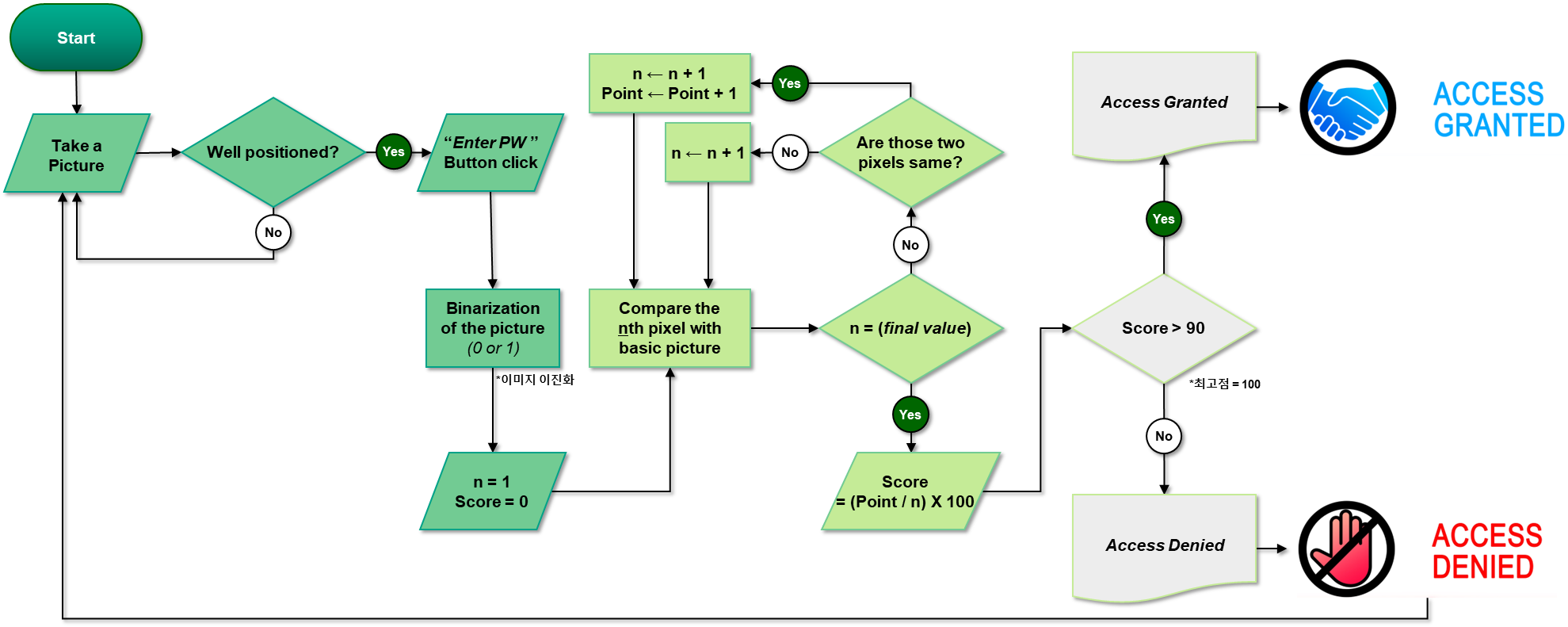


**Supplementary Figure 14.** Algorithm of the smartphone application for 3D double encryption with Fl–Ph MOF-particle-embedded polycaprolactone filament cubes.


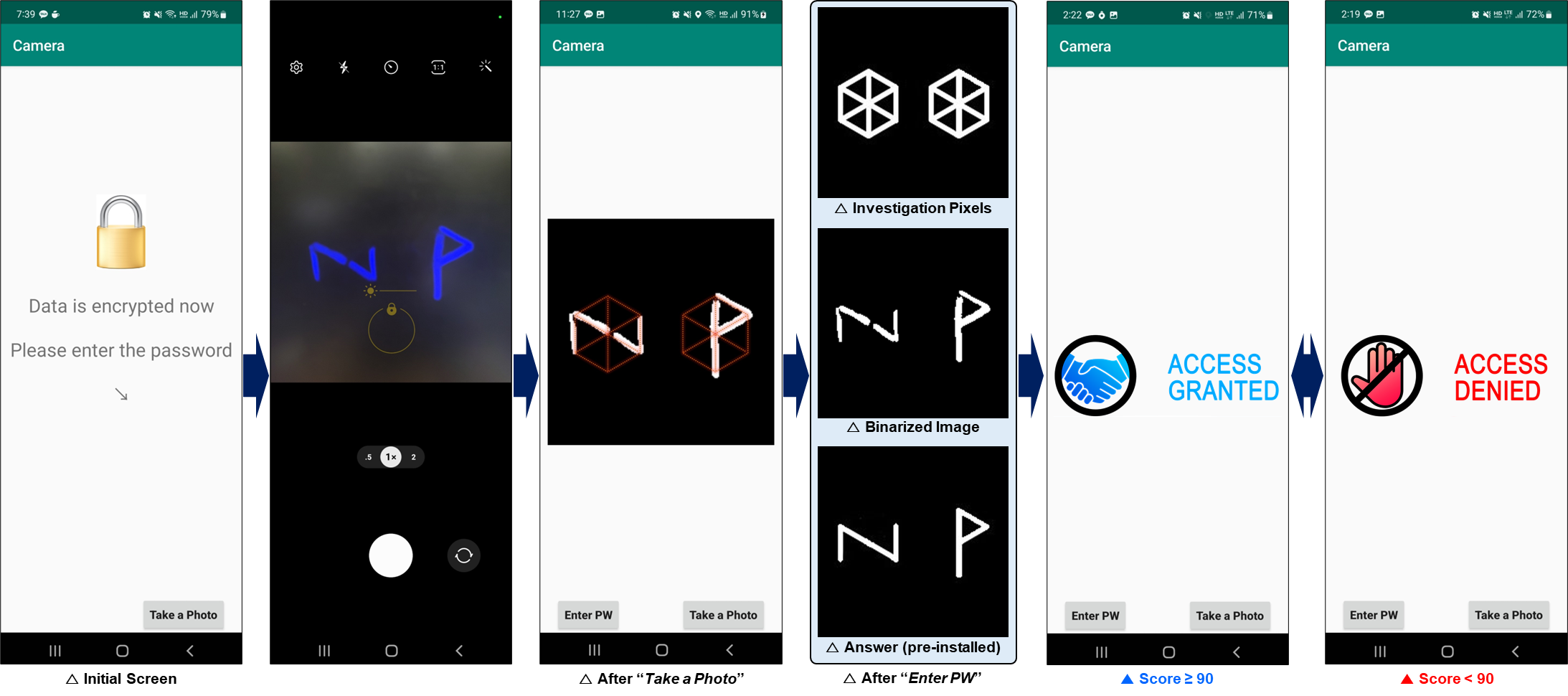


**Supplementary Figure 15.** Real operating screenshots of the smartphone application for 3D encryption.


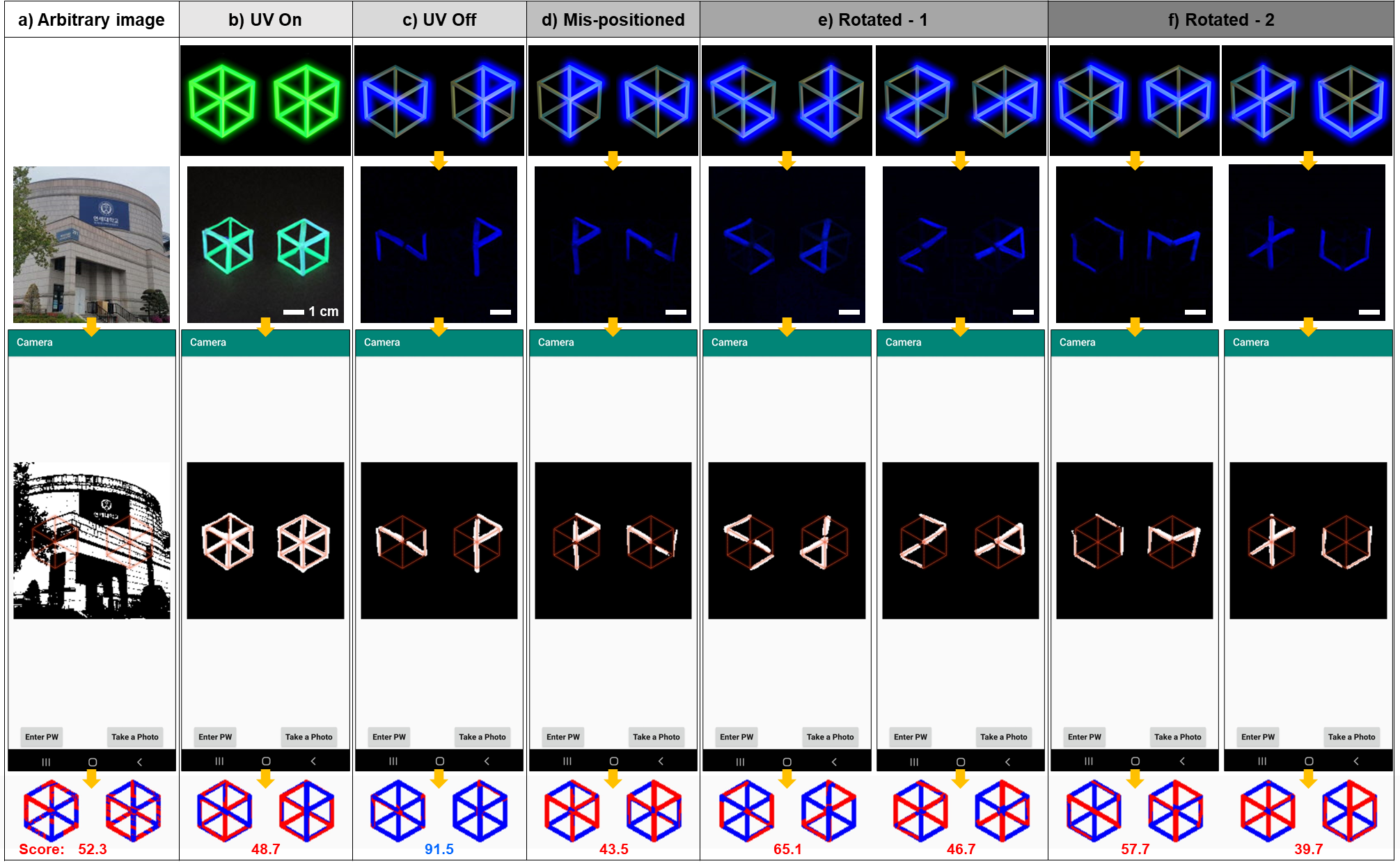


**Supplementary Figure 16.** Photographs of the security level test with (a) an arbitrary picture and 3D cubes with different positions (b) under ultraviolet (UV) on and (c-f) UV off conditions; (c) positioned correctly, (d) mis-positioned, (e) rotated with recognizable alphabets, and (f) rotated with unrecognizable alphabets (top: schematic illustration and photograph captured by smartphone, middle: binarized image, bottom: password comparison result with scores).
